# Hampden Sidney College, Richmond, Va.

**Published:** 1850-05

**Authors:** 


HAMPDEN SIDNEY COLLEGE, RICHMOND, VIRGINIA.

Drs. Maupin and Chamberlayne have been appointed delegates to the convention for revising the Pharmacopœia, and Drs. R. L. Bohannan and C. P. Johnson to the American Medical Association.

